# Review of Dercum’s disease and proposal of diagnostic criteria, diagnostic methods, classification and management

**DOI:** 10.1186/1750-1172-7-23

**Published:** 2012-04-30

**Authors:** Emma Hansson, Henry Svensson, Håkan Brorson

**Affiliations:** 1 Department of Clinical Sciences in Malmö, Lund University, Plastic and Reconstructive Surgery, Skåne University Hospital, Malmö, Sweden; 2 Plastic and Reconstructive Surgery, Skåne University Hospital, SE-205 02, Malmö, Sweden

**Keywords:** Dercum’s disease, Adiposis dolorosa, Adiposalgia, Chronic pain, Adipose tissue, Diagnostic criteria

## Abstract

**Definition and clinical picture:**

We propose the minimal definition of Dercum’s disease to be generalised overweight or obesity in combination with painful adipose tissue. The associated symptoms in Dercum’s disease include fatty deposits, easy bruisability, sleep disturbances, impaired memory, depression, difficulty concentrating, anxiety, rapid heartbeat, shortness of breath, diabetes, bloating, constipation, fatigue, weakness and joint aches.

**Classification:**

We suggest that Dercum’s disease is classified into: I. *Generalised diffuse form* A form with diffusely widespread painful adipose tissue without clear lipomas, II. *Generalised nodular form* - a form with general pain in adipose tissue and intense pain in and around multiple lipomas, and III. *Localised nodular form* - a form with pain in and around multiple lipomas IV. *Juxtaarticular form* - a form with solitary deposits of excess fat for example at the medial aspect of the knee.

**Epidemiology:**

Dercum’s disease most commonly appears between the ages of 35 and 50 years and is five to thirty times more common in women than in men. The prevalence of Dercum’s disease has not yet been exactly established.

**Aetiology:**

Proposed, but unconfirmed aetiologies include: nervous system dysfunction, mechanical pressure on nerves, adipose tissue dysfunction and trauma.

**Diagnosis and diagnostic methods:**

Diagnosis is based on clinical criteria and should be made by systematic physical examination and thorough exclusion of differential diagnoses. Advisably, the diagnosis should be made by a physician with a broad experience of patients with painful conditions and knowledge of family medicine, internal medicine or pain management. The diagnosis should only be made when the differential diagnoses have been excluded.

**Differential diagnosis:**

Differential diagnoses include: fibromyalgia, lipoedema, panniculitis, endocrine disorders, primary psychiatric disorders, multiple symmetric lipomatosis, familial multiple lipomatosis, and adipose tissue tumours.

**Genetic counselling:**

The majority of the cases of Dercum’s disease occur sporadically. A to G mutation at position A8344 of mitochondrial DNA cannot be detected in patients with Dercum’s disease. HLA (human leukocyte antigen) typing has not revealed any correlation between typical antigens and the presence of the condition.

**Management and treatment:**

The following treatments have lead to some pain reduction in patients with Dercum’s disease: Liposuction, analgesics, lidocaine, methotrexate and infliximab, interferon α-2b, corticosteroids, calcium-channel modulators and rapid cycling hypobaric pressure. As none of the treatments have led to long lasting complete pain reduction and revolutionary results, we propose that Dercum’s disease should be treated in multidisciplinary teams specialised in chronic pain.

**Prognosis:**

The pain in Dercum’s disease seems to be relatively constant over time.

## Background

Dercum’s disease is a rare disease listed by Orphanet [1] and by the National Organization for Rare Disorders (NORD) [2]. It is characterised by generalised overweight or obesity, pronounced pain in the adipose tissue and a number of associated symptoms. The pain is chronic (>3 months), symmetrical, often disabling [3] and is resistant to traditional analgesics [4]. The aim of this review was to describe the classification, symptoms and diagnosis of the disease as well as the epidemiology, aetiology, genetic counselling, treatment and prognosis of it.

## Disease name/synonyms

Synonyms of the disease that can be found in the medical literature include Dercum’s disease, Morbus Dercum, adiposis dolorosa, adiposalgia, lipomatosis dolorosa and adipose tissue rheumatism [5].

## Definition and clinical description

**Figure 1:**
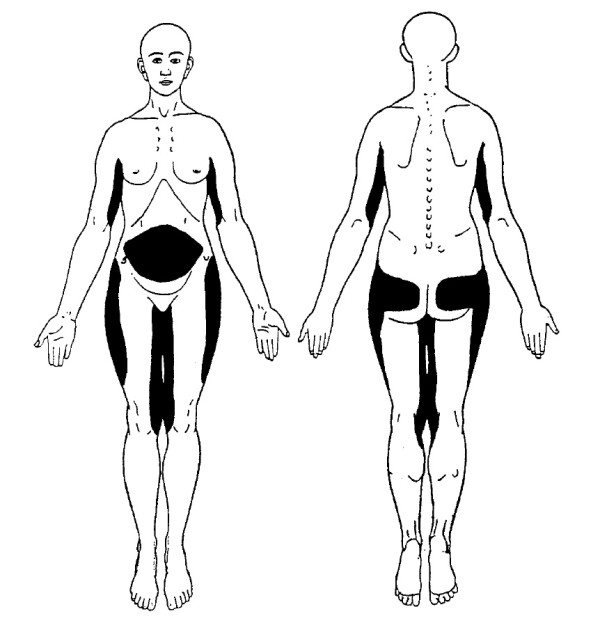
**Typical pain distribution in Dercum’s disease.** Copyright: Håkan Brorson. The figure has previously been published in Läkartidningen [4].

**Figure 2:**
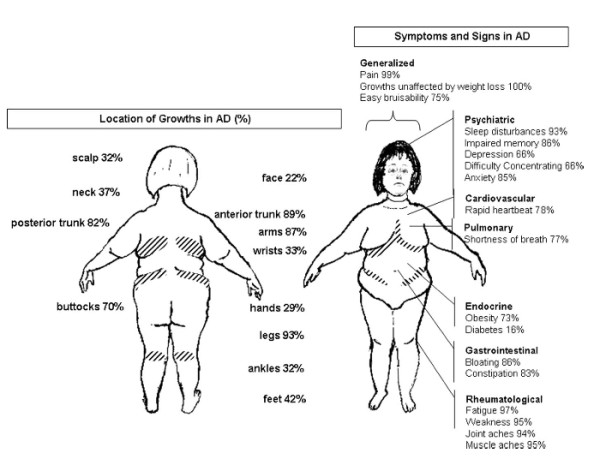
**Cumulative symptoms and signs in 110 individuals with Dercum’s disease according to a questionnaire performed by Herbst and Asare Bediako.** AD: Adiposis dolorosa, which is a synonym to Dercum’s disease. The endocrinologist, 2007 [7]. Copyright: Lippincott Williams and Wilkins. Reprinted with permission.

**Figure 3:**
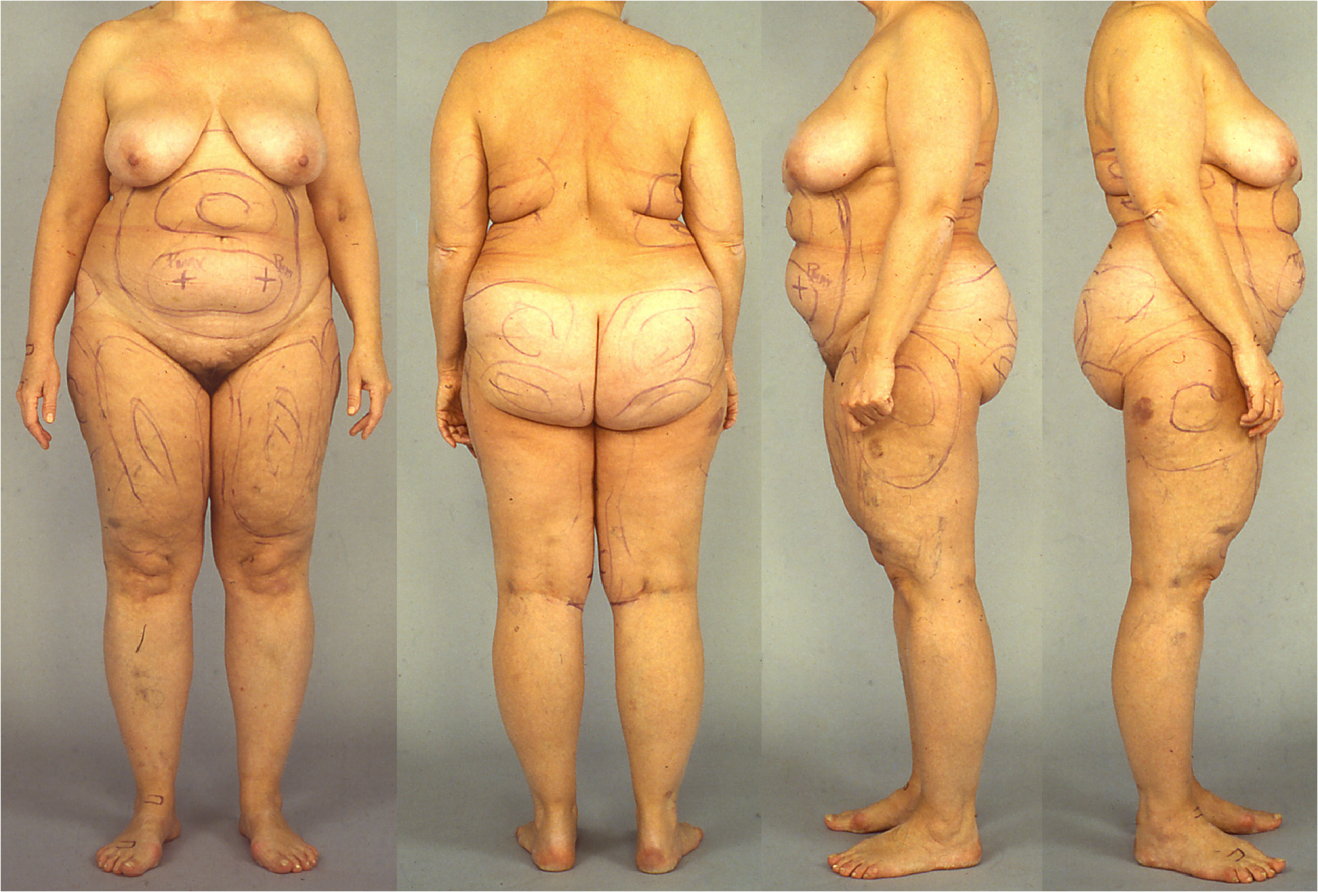
**Patient with Dercum’s disease.** The patient has type I Dercum’s disease, a generalised diffuse form encompassing diffusely widespread painful adipose tissue without clear lipomas. The markings are preoperative markings before liposuction. Written informed consent was obtained from the patient for publication of this report and any accompanying images.

The main symptoms of Dercum’s disease, according to the early reports, are obesity and painful adipose tissue [6]. The most common locations for painful fat and for lipomas are the extremities, the trunk, the pelvic area, and the buttocks (Figure 1). When palpable lipomas exist they vary in size and firmness [7]. The pain is most commonly described by the patients as burning or aching. It can vary from hyperalgesia in the subcutaneous fatty tissue [4] and discomfort on palpation to paroxysmal spontaneous attacks of pain. Based on a questionnaire including 110 individuals with Dercum’s disease Herbst and Asare-Bediako found that the most common locations for painful fat and for lipomas are the extremities, the trunk, the pelvic area, and the buttocks [7] Figure 2. Our experience of pain distribution based on clinical examination of more than 100 patients is depicted in Figure 1 and 3 where the most painful areas are shown. In more general terms upper arms, abdomen, buttocks and thighs are the most common areas. For example, in the upper arm patients have more pain in the medial aspect of the extremity. In the thigh more pain is found medially and laterally than anteriorly and posteriorly. The onset of the condition may be abrupt or indolent [7]. 

In 1901, [Roux and Vitaut 8] proposed four cardinal symptoms of Dercum’s disease:

1. Multiple, painful, fatty masses

2. Generalised obesity

3. Weakness and susceptibility to fatigue (asthenia)

4. Psychiatric manifestations, including emotional instability, depression, epilepsy, confusion, and dementia.

However, it can be discussed which symptoms are cardinal and which are associated. In fact, already in 1927,[ Labbé and Boulin 9] questioned whether the weakness and susceptibility to fatigue and psychiatric manifestations should be classified as cardinal symptoms. They argued that obesity *per se* can induce asthenia, and that it is unclear whether mental disturbances should be included as a cardinal symptom, as they have not been described in all cases of Dercum’s disease [9]. Moreover, in 1910, [Stern 10] remarked that the only two symptoms that are always present in the disease are obesity and painful adipose tissue. Furthermore, in 1930, Gram presented 69 cases of Dercum’s disease and he did not note explicit psychiatric manifestations in any them [11].

Moreover, severe obesity is associated with sleeping disorders [12], which could contribute to the weakness and susceptibility to fatigue experienced by patients with Dercum’s disease. Previously, a clear association between pain obesity, sleep quality and fatigue has been seen in obese patients with fibromyalgia [13]. Furthermore, weakness is seen in for example obese patients with sarcopenia [14].

With regard to the fourth cardinal symptom, psychiatric manifestations, modern research has revealed that pain is a common symptom in depression [15]. Similarly, it has been demonstrated that there is a co-morbidity of chronic pain disease and psychiatric disorders. Post-traumatic stress disorder (PTSD), obsessive-compulsive disorder (OCD), and generalised anxiety disorder (GAD) have been expressly described in a community sample of patients with fibromyalgia [16]. Furthermore, an association between BMI and anxiety and personality disorders has been seen [17]. Hence, the patients’ pain as well as their obesity could contribute to psychiatric manifestations in Dercum’s disease. There are no systematic studies on the social and emotional impact of Dercum’s disease [18].

The four cardinal symptoms have sometimes been used as diagnostic criteria [19-26]. However, as it is unclear which symptoms are cardinal and which symptoms are minor signs in Dercum’s disease, it is unclear which should be used as diagnostic criteria. In addition, there are no laboratory markers for the condition and laboratory tests for inflammatory and autoimmune disease are commonly negative [5,7,27-29]. Altered levels of neuropeptides that have been seen in different pain conditions cannot clearly be seen in Dercum’s disease (Unpublished observations, Hansson, Manjer, Svensson, Åberg, Fagher, Ekman, Brorson). Based on this, we propose the ‘minimal definition’ based on which symptoms are most often part of Dercum’s disease (Table 1):

1. Generalised overweight or obesity

2. Chronic pain (>3 months) in the adipose tissue

**Table 1:** Classification of and diagnostic criteria for Dercum’s disease

**Classification of Dercum’s disease**	
I	Generalised diffuse form
II	Generalised nodular form
III	Localised nodular form
IV	Juxta-articular form
**Diagnostic criteria of Dercum’s disease**	
1	Most often generalised overweight or obesity
2	Chronically painful adipose tissue (>3 months)

Regarding the associated symptoms in Dercum’s disease, only case reports have been published. No study involving medical examinations has been performed in a larger group of patients. However, Herbst and Asare-Bediako have performed a questionnaire including 110 patients with Dercum’s disease. Common symptoms that the patients stated included pain, fatty deposits unaffected by weight loss, easy bruisability, sleep disturbances, impaired memory, depression, difficulty concentration, anxiety, rapid heartbeat, shortness of breath, diabetes, bloating, constipation, fatigue, weakness, joint aches and muscle aches (Figure 2) [7]. 

### Unusual signs and symptoms described

A number of other signs and symptoms have been described in patients with Dercum’s disease. The reported cases are summarised below.

[Haddad et al. 30] reported a case of septic shock secondary to a steatocutanous necrosis in a lipoma in a patient with Dercum’s disease. The patient had a BMI of 46.4 kg/m^2^ and adipose tumours on the abdomen and on the thighs and arms. The lipomas on the thighs had a diameter of circa 30 cm. The necrosis, which was located on one of the thighs, had a diameter of 15 cm and was surrounded by an inflammatory zone of about 30 cm. The necrosis was treated with excision and the sepsis cured with antibiotics. The patient fulfilled the minimal criteria for Dercum’s disease. The necrosis could be caused by a local lymphoedema produced by strangulation of local lymph and blood vessels, and necrosis in the lipomas is thus truly a rare complication in Dercum’s disease.

[Kyllerman et al. 31] described a family with painful lipomatosis. The affected subjects developed associated dysarthria, visual pursuit defects and progressive dystonia. Magnetic resonance imaging revealed bilateral increasing cystic lesions in the basal parts of the putamen. No genetic abnormalities could be detected in the subjects. The condition was interpreted as a Dercum’s disease variant. However, it is unclear whether the patients truly fulfilled the criteria of Dercum’s disease. More data are needed to determine if the cases described indeed had a Dercum’s disease variant.

[Rosenberg et al. 32] reported a case of Dercum’s disease with an unusual distribution of fatty deposits. The adipose tissue infiltrated retroperitoneally, paravesically, and pararectally. The disease started with painful adipose tissue in the left lower extremity. Within a month the pain had spread to the right lower extremity as well. The same year a mass could be palpated along the right side of the rectum. Palpation of the mass produced the same pain in the legs as the patient had experienced spontaneously. An intravenous pyelography and a gravity cystogram revealed hydronephrosis bilaterally and a displaced urinary bladder. A barium enema revealed an elongated sigmoid colon displaced up-ward and to the left. Six months later, the adipose tissue pain had spread to both upper extremities. On examination, an irregular mass, extending from the symphysis pubica to the umbilicus, could be palpated. Neurologically, a positive Babinski’s sign was found on the left side and sensation errors for vibration were noted at and below the iliac crest. During surgery, an adipose tissue tumour was found connected with the peritoneum. The tumour measured 15 by 10 cm and was adherent to the dome of the right wall of the bladder, the right lateral rectal wall, the right ureter and the retroperitoneal tissues. The histological examination revealed normal adipose tissue. The condition was interpreted as Dercum’s disease, where the adipose tissue compressed the urinary bladder, the ureter, the sigmoid colon and the sciatic nerve. In one of the first cases of Dercum’s disease, [Dercum and McCarthy 33] described large masses of fat in the abdomen and around the viscera at autopsy. However, as the patient described by Rosenberg et al. did not have any excessive painful subcutaneous adiposity he did not fulfil the criteria for Dercum’s disease and it is hence unclear if this condition should be classified as a separate entity. Furthermore, contrary to in Dercum’s disease, it was not the fatty masses themselves that were painful, but they caused pressure on other structures and subsequent pain.

[Trentin et al. 34] published a case of Dercum’s disease where the patient developed multiple painful adipose tissue lumps in the breasts. The authors concluded that Dercum’s disease is a rare cause of mastalgia. However, even though many women with Dercum’s disease have painful lipomas in the breast, both idiopathic mastalgia and lipomas are common in the breast and it is undecided if a single painful lipoma of the breast should render the diagnosis Dercum’s disease. On the other hand, it has been discussed if chronic pain syndromes, such as fibromyalgia, might be associated with mastalgia [35].

[Reece et al. 36] noted a patient who developed painful fatty swellings around the neck. Later, a tender lump was excised from the back. The histological examination of the lump revealed normal adipose tissue. The diagnosis of Dercum’s disease was based on the lump found on the back. However, in Dercum’s disease, the head and neck are not affected [4,37], even though this has recently been contradicted by the findings of [Herbst et al. 7]. Most likely the patient described had Madelung’s disease (Lanois-Bensaude syndrome).

[Kawale et al. 38] presented a patient with Dercum’s disease who hade noticed painful thickening of the scalp in bilateral parieto-occipital areas and vertex and pain while combing for more than a year. The pain in the scalp caused headaches and disturbed sleep and daily activities. CT and MRI revealed diffuse thickening of the scalp tissue, but no evidence for other anomalies. However, it is unclear what criteria were used when making the diagnosis Dercum’s disease and hence if the patient truly had Dercum’s disease. The findings could be consistent with encephalocraniocutanous lipomatosis.

[Szypula et al. 28] described a case of Dercum’s disease that was affected with severe hypercholesterolemia and advanced atherosclerosis at the age of 51. However, it is unclear whether there is a link between Dercum’s disease and severe hypercholesterolemia or if the case described merely represent a coexistence of two separate disorders.

Margherita [39] reported a pathological fracture in a patient with Dercum’s disease. However, the origin of the pathological fracture was uncertain. Furthermore, it is uncertain if the patient fulfilled the criteria of Dercum’s disease.

[Tsang et al. 40] noted a case of Dercum’s disease that caused weight loss failure after Roux-en-Y gastric bypass. Eighteen months after the operation the patient was unable to lose weight, despite adherence to behavioural and dietary guidance. Endoscopy performed 15 months after the operation excluded that any complications had occurred. However, it is not stated in the article how the patient was diagnosed with Dercum’s disease and hence we cannot evaluate if the patient truly had the disease. Dercum patients often report that their obesity is refractory to diet and exercise intervention [40]. Nonetheless, this has never been studied.

In brief, it is unclear whether some of the complications described in Dercum’s disease are caused by the condition, or if the occurrence of them is merely coincidental. Furthermore, in a few of the described cases it is unclear if the patients fulfilled the criteria of Dercum’s disease.

## Classification

In 1900, [Giudiceandrea 41] reported a patient and reviewed all the thus far published cases. Based on the results of the review, he proposed the following clinical classification of Dercum’s disease:

I. *Nodular type*. A form with painful lipomas, most commonly on the arms or the legs or on the back or thorax. Sometimes the lipomas occur on multiple locations and occasionally the lipomas form a confluent mass. The nodules are variable in size and painful on palpation.

II. *Diffuse type.* A form with diffusely painful adipose tissue. The pain is symmetric.

III. *Mixed type.* A form with diffusely painful adipose tissue and with painful nodular masses.

In 1901, [Roux and Vitaut 8] modified the classification to the following:

I. *Nodular type.* A form with intense pain in and around multiple lipomas.

II. *Circumscribed diffuse type.* A form with painful folds of fats. The folds are often located on the inside of the knee and/or on the hips.

III. *Generalised diffuse type.* A form with diffusely widespread painful adipose tissue. The most common location of the pain is the extremities and the trunk.

Another classification:

I. *Type I* - juxta-articular

II. *Type II* - diffuse-generalised

III. *Type III* - nodular

is sometimes mentioned [4,27,42]. However, we have not been able to establish who made this classification and when it was compiled. The first time the term juxta-articular Dercum’s disease was used was in 1937 by [Kling 43]. Kling reviewed 112 patients and suggested that juxta-articular Dercum’s disease could be a precursor to the diffuse generalised type of Dercum’s disease*.* Since then, three cases of juxta-articular Dercum’s disease in association with seropositive rheumatoid arthritis have been reported [44,45], and [Weinberger et al. 45] have speculated on whether juxta-articular Dercum’s disease could actually be an extra-articular manifestation of rheumatoid arthritis. Furthermore, Kling has come up with the theory that adipose tissue deposits around the knees might interfere with the blood supply, by pressure on the joint, and result in the development of painful osteo-arthritis [43]. In brief, it is unclear whether the juxta-articular form of Dercum’s disease exists, or if painful localised fat around the joints simply should be designated as the circumscribed diffuse form.

Based on our review of the literature and studies on 111 patients with Dercum’s disease [46-48] we propose that Dercum’s disease should be classified into (Table 1):

I. *Generalised diffuse form:* A form with diffusely widespread painful adipose tissue without clear lipomas.

II. *Generalised nodular form:* A form with general pain in adipose tissue and intense pain in and around multiple lipomas.

III. *Localised nodular form:* A form with pain in and around multiple lipomas

IV. *Juxta-articular form*: A form with solitary deposits of excess fat for example at the medial aspect of the knee

The suggested classification would facilitate differential diagnosis and improve the research on Dercum’s disease.

## Epidemiology

Dercum’s disease most commonly appears between the ages of 35 and 50 years [7,49]. It is five [7] to thirty times [50] more common in women than in men and, originally, Dercum proposed that the condition mainly affects postmenopausal women. However, a recent survey has revealed that 85.7 percent of the included patients developed Dercum’s disease before menopause [7]. The prevalence of Dercum’s disease has not yet been exactly established.

## Aetiology

The aetiology of Dercum’s disease is unknown. However, a number of theories for the aetiology have been suggested. They are summarised below.

### Endocrine dysfunction

Originally, Dercum [6] attributed the disease to an endocrine dysfunction, as he found atrophy of the thyroid gland. Similarly, [Waldorp 51] proposed that the disease is caused by hypophyseal dysfunction. Furthermore, [Winkelman and Eckel 52] reviewed 16 autopsies of patients affected with Dercum’s disease and noted varying abnormalities in different endocrine organs. In their study, the pituitary gland was abnormal in eight of eleven cases examined, the thyroid in twelve cases, the sex glands in nine, the adrenal glands in three and the pancreas in two cases.

However, endocrine involvement was doubted as early as in 1933 [32] and further ruled out in 1952 [53]. In addition, present-day methods have not revealed any endocrine abnormalities [19,22,42,54,55]. For example, [Piementa et al. 54] have demonstrated that hormonal secretion was normal both basally and during a 24 h period in a patient with Dercum’s disease, and no hormonal deficiency could be detected after an integrated pituitary stimulus test. The study suggested that there probably are no alterations of the endocrine glands, as regards the pituitary gland, the adrenal glands, the thyroid and the ovaries, in patients with Dercum’s disease. In another study, a paradoxical increase in growth hormone was seen after an intravenous injection of thyroptropin [56]. The significance of this finding is unclear. In brief, an endocrine dysfunction as the aetiology of Dercum’s disease has little support in the modern literature.

### Nervous system dysfunction

[Dalziel 57] suggested that the autonomous nervous system mediates pain in Dercum’s disease. The theory is supported in that, even though the sympathetic nervous system is efferent, sympathectomy sometimes relieves neuropathic pain, where evidence of damage to neural structures exists [58]. This has been explained by the formation of abnormal connections between autonomic and sensory nerves in the periphery and, as a consequence, abnormal autonomic signalling to the spinal column that might activate pain fibres [57]. However, in conditions where neural damage cannot be found, the effect on pain might be caused by the placebo response [59]. Moreover, patients with Dercum’s disease could have increased sympathetic activity induced by pain. This theory is supported by a study where a patient with Dercum’s disease did not have any vasoconstrictor response to arm and leg lowering. A normalised vasoconstrictor response could be created by lidocaine infusion that is thought to decrease the local or central sympathetic vasoconstrictor tone [60].

Furthermore, visceral pain may be generated by the autonomic nervous system, and factors that induce visceral pain could also have the ability to induce pain in the adipose tissue. Examples of such factors are anoxemia, formation and accumulation of pain-producing substances, traction or compression of vessels, inflammatory states, and necrosis [57]. Nonetheless, any substantial evidence of nervous system dysfunction has never been found in Dercum’s disease and is hence merely a theory.

### Mechanical pressure on the nerves

Pain has been suggested to arise from the stretching of and pressure on nerves by growing fatty masses [23,61]. However, this theory has never been confirmed histopathologically.

### Adipose tissue dysfunction

Another aetiology that has been proposed for Dercum’s disease is a local defect in lipid metabolism [19]. An investigation [19] of fatty acid biosynthesis in two patients with Dercum’s disease suggested that there might be a deficit in the formation of mono-unsaturated fatty acids in subjects affected by the disease. In one of these cases, there was a discrepancy in the formation of long-chain mono-unsaturated fatty acids (16:1 and 18:1) (fatty acids are denoted as number of carbon atoms:number of double bonds) in painful adipose tissue, whereas the formation in the unaffected adipose tissue was normal. In the other case, synthesis of 16:1 fatty acids was detected; on the other hand, the synthesis of 18:1 fatty acids seemed to be completely blocked in painful adipose tissue. However, contradictory findings were revealed in another study, comprised of 13 patients with Dercum’s disease [62]. This investigation showed that the proportion of monounsaturated fatty acids (16:1 and 18:1) was significantly higher in the patients with Dercum’s disease than in the healthy controls. On the other hand, the proportions of saturated (14:0 and 18:0) and some other unsaturated fatty acids (18:3 and 20:1) were increased.

Another finding that supports a local defect in lipid metabolism is corticosteroid-induced juxta-articular Dercum’s disease [63]. A causal relationship between corticosteroids and Dercum’s disease was suggested as the symptoms of Dercum’s disease developed in one case when the patient was temporarily treated with high-dose corticosteroids; the symptoms later resolved when the dosage was reduced. The authors suggested that the effect of corticosteroids on lipid metabolism could have caused the temporary onset of Dercum’s disease.

Pimenta et al. performed an *in vitro* study on normal and painful fat from a patient with Dercum’s disease. The study revealed that the painful adipose tissue had reduced responsiveness to norepinephrine and a lack of response to the anti-lipolytic effect of insulin compared to non-painful adipose tissue [54]. In another *in vitro* study, a sample of painful adipose tissue from a subject affected with Dercum’s disease converted glucose to neutral glycerides at a significantly lower rate than non-painful adipose tissue from the same subject [56].

In addition, one of the studies [62] also revealed that patients with Dercum’s disease have significantly higher fat-cell heat production, compared to obese healthy controls, expressed in μW/g, as well as in pW/cell. The authors speculated on whether this could be explained by higher sympathetic activity due to nociceptive stimuli in the painful adipose tissue. However, one patient in the study had unilateral disease, and adipose cells from the painful side demonstrated lower heat production than cells from the pain-free side. Furthermore, a study [64] performed on 10 patients with Dercum’s disease concluded that women with Dercum’s disease have lower resting energy expenditure on the basis of total body mass (kg) than healthy controls. The relevance of these findings is unclear.

In conclusion, as regards adipose tissue function in Dercum’s disease, the findings are inconclusive and the pathophysiological and clinical significance of the findings are unclear.

### Inflammation

An inflammatory aetiology has been proposed for Dercum’s disease [4,28,65]. However, laboratory markers for inflammation markers, such as erythrocyte sedimentation rate (ESR) and C-reactive protein (CRP), are usually normal in the condition [5,7,19,27-29,39,42,44,50,63,66-68]. However, a few studies have revealed that some patients have elevated levels of CRP and ESR. In A study from 1937, including 112 females, reported that 66% of the patients with Dercum’s disease have been observed to have ESR >15 [43]. A study by [Herbst and Asare-Bediako 7], 33% of the patients with Dercum’s disease had elevated CRP levels and 38% had elevated ESR levels. However, 38% of the patients included in the study had autoimmune disease, such as rheumatoid arthritis and lupus Furthermore, markers for autoimmune disease, such as rheumatoid factor (RF), antinuclear antibodies (ANA), anticardiolipin antibodies (ACA), perinuclear anti-neutrophil cytoplasmic antibodies (pANCA), cytoplasmic anti-neutrophil cytoplasmic antibodies (cANCA) and antibodies against native DNA, are commonly negative in Dercum’s disease [5,27,28,44]. In the study by [Herbst and Asare-Bediako 7], 31% of the patients had positive titres for ANA. However, it is unclear if these patients were among the 38% that had an autoimmune disease.

A study on adipokines in Dercum’s disease has indicated that there is no difference in the levels of tumour necrosis factor (TNF)-α, leptin, adiponectin, plasminogen activator inhibitor-1, interleukin (IL)-1β, IL-8, IL-10, macrophage inflammatory protein (MIP)-1α, and monocyte chemotactic protein (MCP) compared to controls. Nonetheless, significantly lower MIP-1β expression [64] and a trend toward higher levels of IL-13 and lower levels of fractalkine were seen. The authors concluded that the lowered fractalkine levels were logical, since with prolonged release of fractalkine as seen in neuropathic pain, the receptors to which fractalkine binds are upregulated. This suggests that there is shift from fractalkine release to receptor-bound fractalkine. The lower levels of fractalkine found in Dercum’s disease could thus suggest that the substance is receptor-bound. When receptors are occupied by fractalkine, pain and resistance to opioid analgesia are promoted. This is in accordance with the symptoms in Dercum’s disease [64]. A recent investigation of inflammatory signs in adipose tissue of Dercum patients suggests that there is an inflammatory response. However, this response is not more pronounced than that in healthy obese controls. This contradicts inflammation as the aetiology of Dercum’s disease [47].

In brief, there are no uniform findings pointing to an inflammatory aetiology in Dercum’s disease.

### Trauma-induced Dercum’s disease

Two cases of trauma-induced [39,69] Dercum’s disease have been described. The first patient developed a painful fatty tumour, which was very sensitive to pressure and gave rise to much spontaneous pain, after falling on a stone pavement. The painful adipose tissue lingered for a year after the disappearance of the bruises [69]. In the second case, the patient fell down a tree and landed on his right shoulder one year before the onset of Dercum’s disease. No fracture could be detected. One year after the accident, a painful adipose tissue tumour started to grow on his right shoulder. Five years after the injury, an x-ray of the painful shoulder was performed and a humeral fracture that appeared pathological was found. However, the origin of the pathological fracture is unclear [39].

All in all, cases of trauma induced Dercum’s disease seem to occur. However, in some of the cases reported in the literature the evidence is circumstantial.

## Diagnosis (diagnostic criteria and algorithms) and diagnostic methods

The basic diagnostic criteria for Dercum’s disease are (Table 1):

1. Generalised overweight or obesity

2. Chronic pain (>3 months) in the adipose tissue

Diagnosis should be made by systematic physical examination and thorough exclusion of differential diagnoses. Advisably, the diagnosis should be made by a physician with a broad experience of patients with painful conditions and knowledge of family medicine, internal medicine or algology. The diagnosis should only be made when the differential diagnoses have been excluded [70].

Based on our clinical experience and review of the literature we propose the following additional guidelines for diagnosis: Patients who have painful generalised overweight or obesity of the lower extremities should only be diagnosed as lipoedema. If there are isolated painful lipomas or accumulations of fat, the patient should be diagnosed with nodular Dercum’s disease. In cases where the patient fulfils the criteria for fibromyalgia they should primarily be diagnosed with fibromyalgia. Only if the patient also has lipomas should he or she be diagnosed with Dercum’s disease as well. Patients who only have excess fat accumulation in the head, neck region, and upper torso should be diagnosed with Madelung’s disease (Lanois-Bensaude syndrome).

## Differential diagnoses

Many diagnoses have similar symptoms as the symptoms experienced in Dercum’s disease. The diffuse types of Dercum’s disease have traits in common with conditions with general pain:

Fibromyalgia

Lipoedema

Panniculitis

Endocrine disorders encompassing obesity and pain, e.g. Cushing’s syndrome and hypothyroidism

Primary psychiatric conditions encompassing pain, e.g. depression. Especially in combination with obesity.

The nodular type of Dercum’s disease has to be differentiated from conditions that may include lipomas or solitary deposits of excess fat that are sometimes painful:

Multiple symmetric lipomatosis (Madelung’s disease, Lanois-Bensaude syndrome)

Neurofibromatosis type I

Adipose tissue tumours

Multiple endocrine neoplasia I (MEN I)

Myoclonic epilepsy with red ragged fibres (MERRF)

Familial multiple lipomatosis

The similarities and differences of these conditions and Dercum’s disease are briefly presented below.

Fibromyalgia is a condition with widespread muscle pain and a painful response to pressure on at least 11 out of 18 specific tender point sites. It is associated with a range of other symptoms, such as sleep disturbances, fatigue, cognitive disturbances, and depressive symptoms [71]. The aetiology is unknown [75,76] and the demarcation from Dercum’s disease is sometimes arbitrary, especially in cases when the patient is obese.

Lipoedema is characterised by bilateral, symmetric lower extremity enlargement due to subcutaneous deposition of adipose tissue. The patients experience pain on palpation. The condition affects women almost exclusively. Typically, the disorder develops insidiously after puberty and progresses gradually. Moreover, patients with morbid obesity and longstanding lipoedema can develop a secondary mechanical insufficiency of the lymphatic system, producing a lipolymphoedema [72] due to associated difficulties with ambulation, which limits activation of the muscle pump in the lower extremities and subsequently leads to pitting oedema due to inactivity [73].

Panniculitis is a group of inflammatory conditions in which the principal focus is the subcutis. The condition encompasses painful subcutaneous masses and is therefore a possible differential diagnosis to Dercum’s disease. Panniculitis is classified according to which structure it affects: septal panniculitis denotes inflammation in the connective tissue septa, whereas lobular panniculitis refers to inflammation in the fat lobules. Panniculits occurs with or without accompanying vasculitis. Septal panniculitis without vasculitis (erythema nodosum) most commonly manifests itself as tender erythematous nodules on the lower extremity. As some nodules heal, others arise. Normally, all lesions heal without scarring within six weeks. Lobular panniculitis with vasculitis (erythema induratum, nodular vasculitis) presents itself with recurrent, tender, erythematous subcutaneous nodules on the lower extremity. The lesions frequently ulcerate, and they heal with atrophic scars. The condition may continue for several years. A special form of panniculitis is Pfeifer-Weber-Christian disease (idiopathic relapsing febrile lobular non-suppurative panniculitis)[74].

A number of endocrine disorders can cause generalised pain in combination with obesity and psychiatric symptoms. For example, Cushing’s syndrome (corticotropin-independent adrenal hyperfunction) is characterised by aching joints, as well as a gradual onset of obesity, typically located around the face (‘moon face’), back (‘buffalo hump’), and trunk. In addition, the majority of patients suffer from psychiatric manifestations, including irritability, emotional labiality, and depression [75]. Another endocrine disorder, encompassing weight gain and pain in the extremities, is hypothyroidism. If myxoedema develops, oedema of the face, hands and feet are added to the signs [75]. Usually, the patients with endocrine disorders do not have painful adipose tissue.

Lipodystrophies are characterised by selective loss of body fat from different parts of the body. The adipose tissue loss can be limited, and result in well-demarked subcutaneous areas, or extensive, leading to widespread absence of body fat [77]. There are different types of lipodystrophies, both congenital forms, such as Berardinelli-Seip syndrome, and acquired forms, such as Lawrence syndrome and Barraquer-Simons syndrome [78]. On the other hand, localised excess deposition of fat is also a lipodystrophy and comprise most patients that have liposuction for cosmetic reasons. Furthermore, patients who have no pain and with lipoedema-like distribution of fat also belong to the lipodystrophies.

Patients with depression are often diagnosed with chronic pain conditions and vice versa [79]. Both disorders activate common neurocircuitries, for example, the hypothalamic-pituitary-adrenal axis, limbic and paralimbic structures, ascending and descending pain pathways [80], and it is therefore sometimes difficult to determine whether the pain disorder or the psychiatric condition is the primary diagnosis.

Multiple symmetric lipomatosis is principally distinguished by large subcutaneous fatty masses distributed in a symmetrical fashion around the neck, shoulders, upper extremities, trunk, and sometimes intrathoracic. Epidemiologically, the incidence of the disorder is highest in males and is often associated with alcoholism. The majority of the patients are not obese. The fat accumulations are usually not experienced as painful [81]. Sometimes multiple lipomatosis is inherited in an autosomal-dominant way, and is then referred to as familial multiple lipomatosis. The condition can be associated with an A to G mutation at position A8344 of mitochondrial DNA [82]. The fatty masses in multiple symmetric lipomatosis are not painful and it is hence possible to differentiate from Dercum’s disease. Multiple symmetric lipomatosis is a separate entity from familial multiple lipomatosis.

Some patients with neurofibromatosis type 1 (von Recklinghausen’s disease) develop subcutaneous neurofibromas that cause pain and neurological symptoms [83]. Neurofibromas are pathohistologically distinct from lipomas.

Benign adipose tissue tumours (solitary/multiple) are subcutaneous tumours composed of well-differentiated adipocytes. There are a number of varieties of lipomas, some of which are frequently painful [75]. Malignant cutaneous adipose tissue neoplasms are exceptionally rare and are usually not experienced as painful. Such tumours include cutaneous angiolipoleiomyoma (‘angiomyolipoma’), adenolipoma of the skin, cutaneous spindle cell/pleomorphic lipomas, and liposarcomas [84]. Tumours are normally easily differentiated from Dercum’s disease, as they are not painful.

Patients with multiple endocrine neoplasia I (MEN 1) sometimes exhibit several subcutaneous tumours, including multiple lipomas, which are, however, not painful [85]. MEN 1 is easily differentiated from Dercum’s disease as the lipomas in MEN 1 are not painful.

Myoclonic epilepsy with ragged red fibres (MERRF), an inherited disease of the mitochondria, is sometimes accompanied by multiple symmetric lipomatosis. The most common cause is a mutation in position 8344 of the tRNA gene of mitochondrial DNA [86]. MERRF is easily differentiated from Dercum’s disease, as the lipomas in MERRF are not painful.

In conclusion, the differential diagnosis is a challenge principally in cases where diffuse Dercum’s disease is one of the tentative diagnoses. All differential diagnoses have to be excluded before Dercum’s disease can be diagnosed.

## Genetic counselling

The majority of the cases of Dercum’s disease occur sporadically. We have found five reports on the inheritance of the disorder. The studies have suggested that Dercum’s disease might be an autosomal dominant disorder with variable expression [18,49,87-89]. A gender factor has been proposed as the affected women were markedly obese and experienced considerable pain, whereas the expression of the disease in the affected men was so mild that it would not have been noticed if a very thorough physical examination had not been performed [88]. The possible genetic heterogeneity could explain why the disease has been reported in females with a frequency that is up to 30 times greater than in males [50]. Furthermore, it has been suggested that some cases of Dercum’s disease might actually be an extreme expression of familial multiple lipomas [31,87]. This implies that some of the affected men could have been given the diagnosis familial multiple lipomas instead of Dercum’s disease, as their symptoms are so discreet [49].

In addition, studies have revealed that the A to G mutation at position A8344 of mitochondrial DNA, which is sometimes associated with familial multiple lipomas, cannot be detected in patients with Dercum’s disease [31,87]. Similarly, HLA (human leukocyte antigen) typing, which has been performed in a family of five patients affected by Dercum’s disease [54], has not revealed any correlation between typical antigens and the presence of the condition.

## Management including treatment

Few convincing large studies on the treatment of Dercum’s disease have been conducted. Most of the different treatment strategies that exist are based on case reports. The described treatments are summarised below.

### Liposuction and surgical treatment

A number of patients treated with liposuction have been presented [46,70]. In all cases, suction-assisted liposuction was used. The ‘dry’ technique was applied by all investigators, with the exception of one, who used the tumescence technique with 0.1 percent prilocarpine solution. The mechanism behind pain relief following liposuction is thought to be that nerve plexuses in the adipose tissue are destroyed [57]. However, it is unlikely that direct nerve destruction alone explains the pain reduction seen following liposuction [48]. Furthermore, the reduction in pain in Dercum’s disease persisted longer than sensitivity loss normally seems to persist after liposuction in healthy patients [46].

Lipectomy has been tried in a few cases[23,25,43,50,61,67,90,91]. The treatment was effective, but the pain returned in all cases but one.

### Traditional analgesics

The pain in Dercum’s disease is often refractory to analgesics and to non-steroidal anti-inflammatory drugs (NSAIDs) [44,56,67,68,92-98]. However, this has recently been contradicted by the findings of [Herbst et al. 7]. They reported that the pain diminished in 89% of patients (n=89) when treated with NSAIDs and in 97% of patients when treated with narcotic analgesics (n=37). Nonetheless, the dosage required and the duration of the pain relief were not precisely stated in the article.

### Lidocaine

An early report from 1934 showed that intralesional injections of procaine (Novocain®) relieved pain in six cases [99]. More recently, other types of local treatment of painful sites with lidocaine patches (5%) (Lidoderm®) [29,100] or lidocaine/prilocaine (25 mg/25 mg) cream (EMLA®) [101] have shown a reduction of pain in a few cases.

In the 1980s, treatment with intravenous infusions of lidocaine (Xylocaine®) in varying doses was reported in nine patients [21,22,27,56,60,92,97,102]. The resulting pain relief lasted from 10 hours [21] to 12 months [92]. In five of the cases, the lidocaine treatment was combined with mexiletine (Mexitil®), which is a class 1B anti-arrhythmic with similar pharmacological properties as lidocaine [27,36,42,102].

The mechanism by which lidocaine reduces pain in Dercum’s disease is unclear. It may block impulse conduction in peripheral nerves [21], and thereby disconnect abnormal nervous impulse circuits [102]. Nonetheless, it might also depress cerebral activity that could lead to increased pain thresholds [21]. [Iwane et al. 21] performed an EEG during the administration of intravenous lidocaine. The EEG showed slow waves appearing 7 minutes after the start of the infusion and disappearing within 20 minutes after the end of the infusion. On the other hand, the pain relief effect was the greatest at about 20 minutes after the end of the infusion. Based on this, the authors concluded that the effect of lidocaine on peripheral nerves most likely explains why the drug has an effect on pain in Dercum’s disease. In contrast, [Atkinson et al. 92] have suggested that an effect on the central nervous system is more likely, as lidocaine can depress consciousness and decrease cerebral metabolism [92]. In addition, [Skagen et al. 60] demonstrated that a patient with Dercum’s disease lacked the vasoconstrictor response to arm and leg lowering, which indicated that the sympathicus-mediated local veno-arteriolar reflex was absent. This could suggest increased sympathetic activity. An infusion of lidocaine increased blood flow in subcutaneous tissue and normalised the vasoconstrictor response when the limbs were lowered. The authors suggested that the pain relief was caused by a normalisation of up-regulated sympathetic activity.

### Methotrexate and infliximab

One patient’s symptoms were improved with methotrexate and infliximab (Remicade®) [103]. However, in another patient with Dercum’s disease, the effect of methotrexate was discreet [18]. The mechanism of action is unclear. Previously, methotrexate has been shown to reduce neuropathic pain caused by peripheral nerve injury in a study on rats [104].

The mechanism in the rat study case was thought to be a decrease in microglial activation subsequent to nerve injury [104]. Furthermore, a study has shown that infliximab reduces neuropathic pain in patients with central nervous system sarcoidosis. The mechanism is thought to be mediated by tumour necrosis factor inhibition [105].

### Interferon α-2b

Two patients were successfully treated with interferon α-2b [106]. The authors speculated on whether the mechanism could be the antiviral effect of the drug, the production of endogenous substances, such as endorphins, or interference with the production of interleukin-1 and tumour necrosis factor. Interleukin-1 and tumour necrosis factor are involved in cutaneous hyperalgesia.

### Corticosteroids

A few patients noted some improvement when treated with systemic corticosteroids (prednisolone) [55,93,107], whereas others experienced worsening of the pain [63]. [Weinberg et al. 45] treated two patients with juxta-articular Dercum’s disease with intralesional injections of methylprednisolone (Depo-Medrol®). The patients experienced a dramatic improvement.

The mechanism for the pain-reducing ability of corticosteroids in some conditions is unknown. One theory is that they inhibit the effects of substances, such as histamine, serotonin, bradykinin, and prostaglandins [108]. As the aetiology of Dercum’s disease is probably not inflammatory, it is plausible that the improvement some of the patients experience when using corticosteroids is not caused by an anti-inflammatory effect.

### Calcium-channel modulators

Several calcium-channel modulators have been tried; for instance, pregabalin (Lyrica®) (anticonvulsant) [5] and oxacarbazepine (Trileptal®) (anticonvulsant) [42,66,109].

Calcium-channel modulators inhibit the activation of neuronal calcium channels and thereby inhibit the release of substances, such as excitatory amino acids, that are necessary for central sensitisation. These drugs are used to treat neuropathic pain [110]. Several of these substances have a broad range of pharmacological actions and other mechanisms beyond calcium-channel modulation that might contribute to their effectiveness in treating neuropathic pain [110].

### D-thyroxine

D-thyroxine (Levaxine®) has not given any symptomatic relief in Dercum’s disease [62]. This further supports that the thyroid gland is not involved in the pathogenesis of the disease.

### Rapid cycling hypobaric pressure

A pilot study [111], including 10 patients, has suggested that rapid cycling hypobaric pressure might decrease pain patients with Dercum’s disease. Different mechanisms have been proposed for the effect: pneumatic displacement of fluid, that is, a decrease in tissue oedema, improved blood flow through intermittent compression and an increase in arterial oxygen saturation and an decrease in hypoxia in the painful tissue [111].

## Prognosis

There has been little research conducted on the natural history of Dercum’s disease, but case reports have suggested that the pain might be aggravated over time [18]. However, this is not clearly supported in a recent study with a five year follow-up. In that study, the average pain was relatively constant over five years (about 5/10 on a visual analogue scale). On an individual level some patients experienced less pain after five years and some more [46].

## Unresolved questions

The diagnostic criteria for Dercum’s disease have never been validated. The Classification and Response Criteria Sub-committee, supported by the American College of Rheumatology, have developed recommendations for the development and validation of criteria sets [112]. According to these recommendations, criteria should be developed using appropriate consensus methodology, and all of the criteria should be reliable, that is, they should be stable when there is no clinical change, precise in their measurement, easy to measure and clinically sensible, and differentiate the condition from other conditions[112]. Further work is needed to validate the diagnostic criteria of Dercum’s disease, according to the recommendations of the American College of Rheumatology.

The prevalence of Dercum’s disease has not yet been exactly established and the prognosis is described differently in different reports.

The aetiology of Dercum’s disease is unknown. Syndromes with unclear aetiology and no objectively observable abnormalities are sometimes refereed to as functional somatic syndromes and include, for example, irritable bowel syndrome, non-ulcer dyspepsia, premenstrual syndrome, chronic pelvic pain, fibromyalgia, chronic fatigue syndrome, tension headaches, temporomandibuar joint dysfunction, globus syndrome and multiple chemical sensitivity. These syndromes often affect females, are negatively affected by stress, demonstrate co-existing emotional disorders, have a similar prognosis and respond to different treatments in a similar way [113]. At least diffuse Dercum’s disease may qualify as a functional somatic syndrome, as it overlaps with some of the above mentioned conditions and patients with Dercum’s disease often meet diagnostic criteria for one or several of the functional somatic syndromes. Patients with Dercum’s disease and functional somatic syndromes share non-symptomatic characteristics, that is, they are predominantly women, they often have emotional disorders and difficulties in doctor-patient relationship [113]. This suggests that more general strategies should be developed for the management of Dercum’s disease. Presently, patients in these domains of disease are probably best treated by more general strategies in multidisciplinary teams specialised in chronic pain [113]. Furthermore, this kind of treatment has been used successfully in patients with for example fibromyalgia [114].

## Conclusion

The diagnostic criteria of Dercum’s disease are proposed to be generalised overweight or obesity and painful adipose tissue. However, the diagnostic criteria need to be validated. The disease is preferably classified as: I. *Generalized diffuse form* - a form with diffusely widespread painful adipose tissue, II. *General nodular form* - a form with general and intense pain in and around multiple lipomas, III. *Localized nodular form* - a form with pain in and around multiple lipomas, and IV. *Juxta-articular form* - a form with solitary deposits of excess fat for example at the medial aspect of the knee. The aetiology of Dercum’s disease is unknown and there is hence no good treatment available. Presently, patients with Dercum’s disease are probably best treated by more general strategies in multidisciplinary teams specialised in chronic pain. This kind of treatment has been used successfully in patients with for example fibromyalgia.

## Competing interests

The authors declare that they have no competing interests.

## Authors’ contribution

EH has performed the review and written the manuscript. HS and HB have contributed to the final version of the manuscript. All authors read and approved the final version of the manuscript.

## Authors’ information

Emma Hansson (MD, MA, PhD) is a resident in plastic surgery. Henry Svensson (MD, PhD) and Håkan Brorson (MD, PhD) are senior consultants and professors in plastic surgery.
